# Deciphering the triad of infection, immunity and pathology

**DOI:** 10.7554/eLife.72379

**Published:** 2021-09-01

**Authors:** Frederik Graw

**Affiliations:** BioQuant (Center for Quantitative Biology) at Heidelberg University Heidelberg Germany

**Keywords:** influenza virus, mathematical model, lung injury, lung inflammation, disease, cd8 t cells, Virus

## Abstract

The factors which drive and control disease progression can be inferred from mathematical models that integrate measures of immune responses, data from tissue sampling and markers of infection dynamics.

**Related research article** Myers MA, Smith AP, Lane LC, Moquin DJ, Aogo R, Woolard S, Thomas P, Vogel P, Smith AM. 2021. Dynamically linking influenza virus infection kinetics, lung injury, inflammation, and disease severity. *eLife* **10**:e68864. doi: 10.7554/eLife.68864

A fever, a cough, a splitting headache… Being sick often comes with tell-tale signs which worsen as the disease progresses and tissues become damaged. These symptoms result from complex interactions between the infecting pathogen, the inflammation process, and the response from the immune system. Tracking these mechanisms and how they interact, as well as identifying which factors determine when the disease recedes or progresses, is essential for establishing better treatment strategies.

In this effort, a more refined understanding of infection and immune responses has emerged from combining experimental and clinical measurements with mathematical models (Perelson, 2002). However, it is still difficult to link tissue pathology and disease severity with viral load or immune cell counts, which respectively measure the amount of virus and of certain immune actors in the body. Now, in eLife, Amber Smith and colleagues at St. Jude Children’s Research Hospital, the University of Tennessee Health Science Center and the Washington University School of Medicine – including Margaret Myers and Amanda Smith as joing first authors – report how viral infection, counteracting immune responses and lung pathology interact as mice fight off influenza A (Myers et al., 2021).

First, the team tracked how viral load and the number of CD8+ T cells, an important immune actor that helps to clear infected cells, progressed over time. In combination with mathematical models, these measurements allowed Myers et al. to estimate several parameters that reflect the pace at which the virus replicates, the strength of the immune response, and the interactions between these processes. While this had already been achieved in previous studies (e.g. Baccam et al., 2006), Myers et al. also analyzed the anatomy of the lung tissue over time, assessing the damage caused by infection and inflammation as well as how much the organ eventually regenerates.

Then, the team compared these data to values from their mathematical model that described viral load and CD8+ T cells counts, thereby linking viral load dynamics and specific immune responses to disease pathology and severity (Figure 1). In particular, the analysis shed light on how the relative number of immune cells correlates with the level of lung tissue cleared from the virus and, thus, the mice’s ability to recover from infection. These quantitative relationships could help to assess how well the virus is controlled within tissues simply by relying on easily accessible markers that are, for example, present in the blood. This would reduce the need for invasive tissue samples.

**Figure 1.**
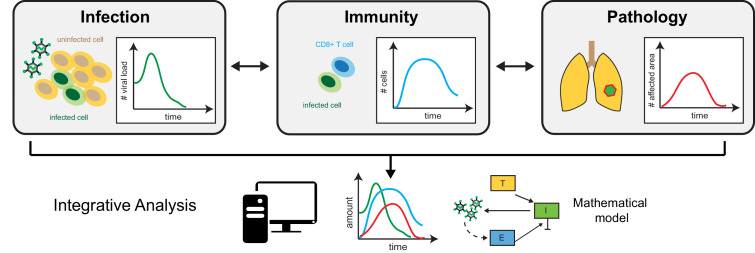
The triad of infection, immunity and disease pathology. Separate data, such as viral load (left) – the quantity of virus present in a specific volume of fluid – immune cell counts (middle) and histological assessment of tissue sections (right) provide information on the dynamics of infection, immune responses and disease pathology. Mathematical modelling that integrates these measurements makes it possible to assess how the individual processes are connected, and to identify relevant prognostic markers that allow prediction of disease progression.

Individual molecular processes and specific aspects of viral replication can be studied extensively within in vitro cell culture systems. However, the full triad of infection, immunity and especially tissue pathology can only be reliably assessed within conditions that are physiologically relevant (Fackler et al., 2014). Indeed, simple cell culture systems insufficiently address the impact tissue structure can have on infection dynamics, immune activation and clearing mechanisms (Fackler et al., 2014; Imle et al., 2019).

Myers et al. used frequent samples and histological analyses to infer how infected tissues change over time. Yet, imaging technologies may continue to improve so that it becomes possible to observe the interactions between host and pathogen within tissues in real-time (Coombes and Robey, 2010). These approaches could help to investigate whether quantitative relationships as highlighted by Myers et al. also play a role in other infections and in other tissues. The expanding field of organoids – whereby simple, miniature organs are grown in the laboratory – also represents a promising step towards understanding how cells interact within structured, tissue-related environments (Gosselin et al., 2018; Bar-Ephraim et al., 2020). Combined with new technologies such as single-cell sequencing methods (Triana et al., 2021; Youk et al., 2020), these approaches will help to determine the molecular processes that govern disease progression, and how these might differ between patients.

Despite these new experimental and diagnostic technologies, data-driven mathematical modeling and analytical methods will continue to fulfil a key role for deciphering the interplay between infection, tissue pathology and disease severity. Using these models makes it possible to integrate different types of measurements from various places and times, and to disentangle the contributions of individual processes to the infection dynamics. It is only by understanding exactly how individual processes interact over time that scientists will be able to find and validate prognostic markers which predict disease progression.
